# Reduced insulin receptor signaling in retinal Müller cells cultured in high glucose

**Published:** 2013-04-05

**Authors:** Youde Jiang, Jayaprakash Pagadala, Duane Miller, Jena J. Steinle

**Affiliations:** 1 Department of Ophthalmology, University of Tennessee Health Science Center, Memphis, TN; 2 Department of Anatomy & Neurobiology, University of Tennessee Health Science Center, Memphis, TN; 3 Department of Pharmaceutical Sciences, University of Tennessee Health Science Center, Memphis, TN

## Abstract

**Purpose:**

To measure key proteins involved in insulin resistance in retinal Müller cells.

**Methods:**

Cells known as retinal Müller cells were cultured in normal (5 mM) or high glucose (25 mM) to mimic a diabetic condition. Cells were treated with 50 nM Compound 49b, a novel β-adrenergic receptor agonist. Additional cells were treated with small interfering RNA (siRNA) against protein kinase A or cyclic adenosine monophosphate (cAMP) responsive element binding protein (CREB). Western blotting or enzyme-linked immunosorbent assay (ELISA) measurements were made for protein changes in TNFα, suppressor of cytokine signaling 3, insulin receptor substrate 1 (IRS-1), insulin receptor (IR), Akt, and cell death proteins (Fas, fas ligand, cytochrome C, Bax, cleaved caspase 3, and Bcl-xL).

**Results:**

Hyperglycemia significantly increased TNFα and suppressor of cytokine signaling 3 levels. This was associated with increased phosphorylation of IRS-1^Ser307^ and IR^Tyr960^, with decreased phosphorylation of IR^Tyr1150/1151^ and Akt^Ser473^. The reduced insulin receptor and Akt phosphorylation led to a significant increase in proapoptotic proteins. Compound 49b reversed the loss of Akt and IR^Tyr1150/1151^ phosphorylation, reducing Müller cell apoptosis.

**Conclusions:**

Hyperglycemia-induced TNFα levels promote insulin resistance in retinal Müller cells, noted through increased phosphorylation of IRS-1^Ser307^ and IR^Tyr960^. The dysfunctional insulin signaling increases apoptosis of retinal Müller cells, which is blocked through treatment with Compound 49b. Taken together, β-adrenergic receptor agonists may protect retinal Müller cells through maintenance of normal insulin receptor signaling.

## Introduction

Diabetic retinopathy is the leading cause of blindness in working-age adults, with rates expected to rise exponentially due to increasing rates of diabetes. While a large number of hypotheses have been provided as to the potential causes of retinal damage due to diabetes, few have translated into new therapies. One potential reason for this discrepancy is that any drug must produce a positive effect on multiple cell types in the retina. It is probable that the cells of the retina respond differently to high glucose, all working to preserve homeostasis. We have previously reported that a novel β-adrenergic receptor agonist, Compound 49b, was effective in preventing apoptosis of retinal endothelial cells (RECs) through activation of insulin-like growth factor binding protein 3 (IGFBP-3) and reducing TNFα levels [1]. We defined Compound 49b as a β-adrenergic receptor agonist, as it significantly increased protein kinase A (PKA) levels and was based on the chemical structure of isoproterenol with the addition of an N-substituent *3,4,5-trimethoxylphenethyl* ring [1]. Decreased apoptosis after Compound 49b was also observed in retinal lysates from diabetic rats. While these findings are important, the retinal vasculature may be one of the last parts to undergo apoptosis or degenerative changes due to hyperglycemia, since degenerate capillaries and pericyte ghosts are not observed until 6–8 months after diabetes is induced in rodents [2,3]. Others have reported that neuronal and Müller cells respond much earlier to high-glucose conditions [4-6]. Increased glial fibrillary acidic protein (GFAP) levels in retinal Müller cells of diabetic animals have been reported within 6–8 weeks of diabetes induction [6]. Because Müller cells are protective of retinal endothelial cells [7], the ideal novel drugs should protect both RECs and retinal Müller cells.

One of the key responses of Müller cells to hyperglycemia is an increase in cytokines, specifically IL-1β [8] and TNFα [9]. In fact, a recent study suggested that RECs are more responsive to cytokines produced by other retinal cells, compared to high glucose alone [10]. One of the key cell types evaluated in that study was human Müller cells. Retinal Müller cells likely respond to the stressor of high glucose with increased GFAP and cytokine levels, thus affecting retinal physiology.

Altered insulin receptor signaling can greatly affect retinal homeostasis, as activation of the insulin receptor is antiapoptotic [11,12]. In the normal retina, activation of the insulin receptor with insulin will increase phosphorylation of insulin receptor substrate-1 (IRS-1) or IRS-2, leading to phosphorylation and activation of Akt. We have previously reported that streptozotocin-induced diabetes in the rat retinas led to decreased insulin receptor and Akt phosphorylation, which was associated with increased apoptotic levels [1,13]. Further, we have shown that knockdown of insulin receptor substrate-1 (IRS-1) results in increased apoptosis of retinal Müller cells [14]; there are similar results obtained when TNFα is knocked down [15]. Taken together, these results suggest that diabetes decreases insulin receptor signaling in the retina, which likely involves TNFα levels.

The key question for retinal Müller cells is how TNFα might regulate insulin receptor signaling. Two potential pathways have emerged from work on adipocytes and myocytes to demonstrate that TNFα can inhibit normal insulin signal transduction. First, increased TNFα levels leads to phosphorylation of IRS-1^Ser307^, but the impact of this specific phosphorylation has not been investigated in the retina. For the insulin signal to be propagated to Akt, leading to the antiapoptotic actions of Akt, insulin must interact with insulin receptor substrates 1–4. We have identified IRS-1 as key to this interaction in retinal Müller cells [14,15]. Second, TNFα can activate the suppressor of cytokine signaling 3 (SOCS3) pathway [16-18]. We have previously reported that TNFα can induce SOCS3 to promote REC apoptosis [19]. SOCS3 can repress insulin signal transduction through phosphorylation of the insulin receptor on tyrosine 960, which blocks formation of the complex between the insulin receptor and IRS-1. Alternatively, SOCS3 can promote ubiquinitization of IRS-1, thus eliminating IRS-1 from the complex [16]. In either case, TNFα activation of SOCS3 leads to inhibition of normal insulin signal transduction.

While we have shown that knockdown of IRS-1 or TNFα can protect retinal Müller cells from apoptosis [14,15], we did not fully tease out the insulin resistance pathways activated by TNFα. We have shown that a selective β-2-adrenergic receptor agonist, salmeterol, can protect the retina; however, since RECs do not possess β-2-adrenergic receptors [20], the goal is to develop a novel drug that can prevent insulin resistance signaling in both RECs and Müller cells. In this work, we tested the hypothesis that Compound 49b, a novel β1- and β2-adrenergic receptor agonist, could significantly reduce TNFα levels to promote normal insulin signal transduction in retinal Müller cells.

## Methods

### Müller cell culture

The rMC-1 (rat Müller cells) cells were kindly provided by Dr. Vijay Sarthy (Northwestern University). Müller cells were grown in normal (5 mM) or high glucose (25 mM) Dulbecco’s modified Eagle’s medium (DMEM) medium supplemented with 10% fetal bovine serum and antibiotics. Once cells reached 80% confluence, cells were starved (no FBS) overnight (~16 h) in high-glucose medium and treated with 50 nM Compound 49b the following morning. Twenty-four hours later, cells were scraped into lysis buffer for enzyme-linked immunosorbent assay (ELISA) or western blot analyses. Additional Müller cells were grown in both glucose conditions and were transfected with PKA small interfering RNA (siRNA) or cyclic adenosine monophosphate (cAMP) responsive element binding protein (CREB) siRNA (Dharmacon, Lafayette, CO) for 24 h before treatment with 50 nM Compound 49b for an additional 24 h.

### ELISA analyses

The PKA Activity Assay (Millipore, Bilerica, MA), TNFα ELISA (ThermoScientific, Pittsburgh, PA), cleaved caspase 3 ELISA (Cell Signaling, Danvers, MA), and an IGFBP-3 ELISA (R&D Systems, Littleton, CO) were done according to manufacturers’ instructions. For the PKA Activity Assay and cleaved caspase 3 ELISA, equal protein concentrations were loaded into each well to allow for analyses based on the optical density measurement.

### Western blotting

**Figure 1:**
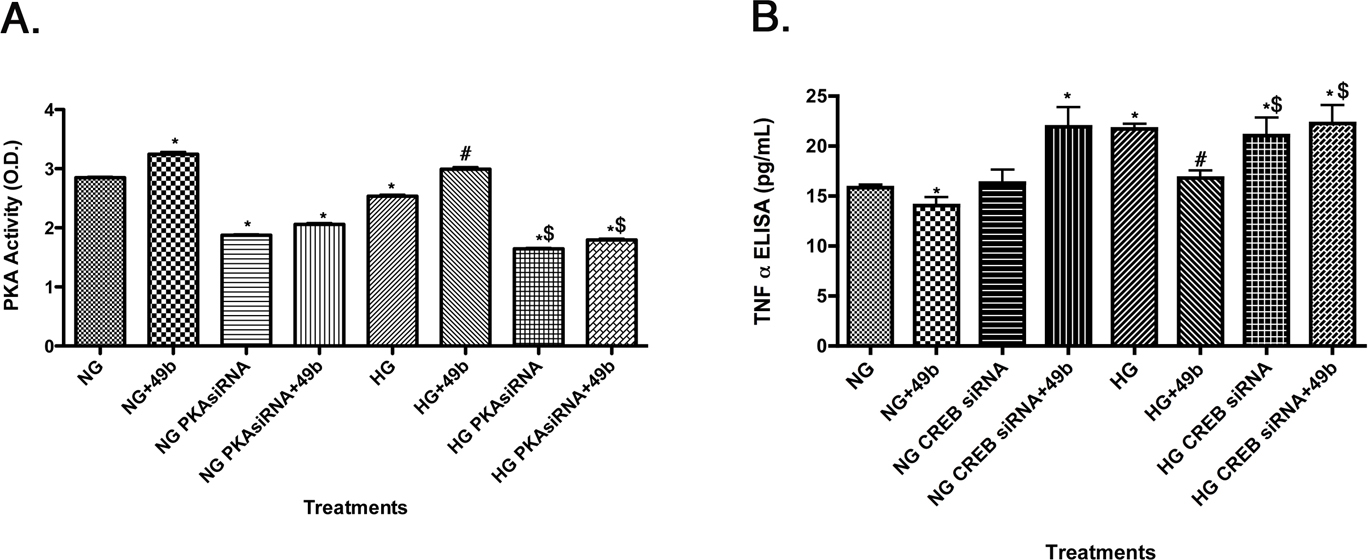
Figure presents ELISA results for retinal Müller cells cultured in normal glucose (5 mM, NG) or high glucose (25 mM, HG) and treated with Compound 49b (50 nM) or PKA siRNA or CREB siRNA. **A:** These ELISA results for PKA activity demonstrate that PKA siRNA is effective in reducing PKA activity levels. **B** shows ELISA results for TNFα after Compound 49b treatment with or without CREB siRNA, to demonstrate that Compound 49b regulates TNFα through PKA and CREB action. *p<0.05 versus NG, #p<0.05 versus HG, $p<0.05 versus HG+49b. n=4 for all groups. Data are mean±SEM.

**Figure 2:**
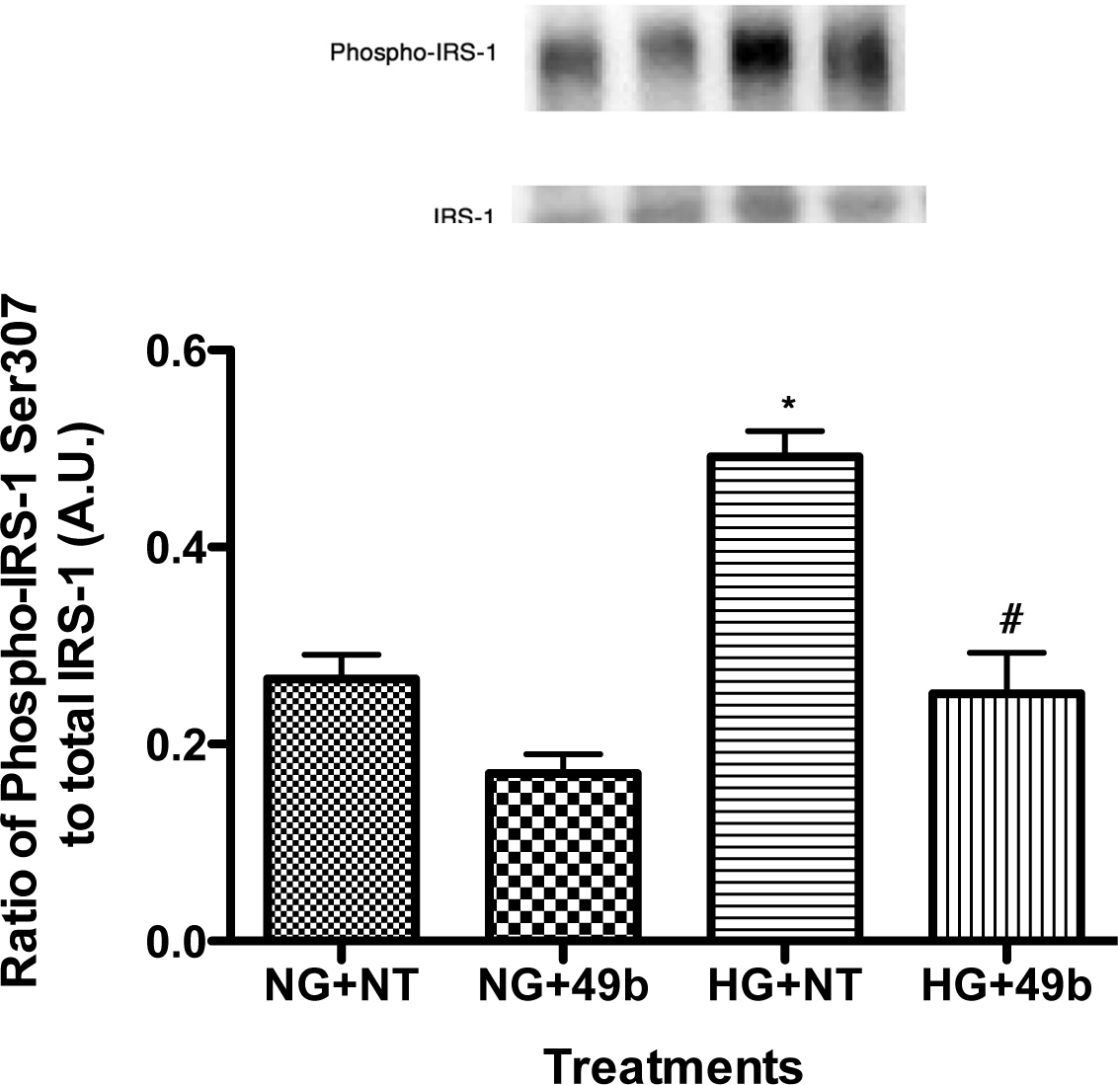
Data are western blot results for phosphorylated IRS-1^Ser307^ to total IRS-1 on retinal Müller cells cultured in normal glucose (5 mM, NG) or high glucose (25 mM, HG) and treated with Compound 49b (50 nM). A representative blot is shown. *p<0.05 versus NG, #p<0.05 versus HG, n=4 for all groups. Data are mean±SEM.

**Figure 3:**
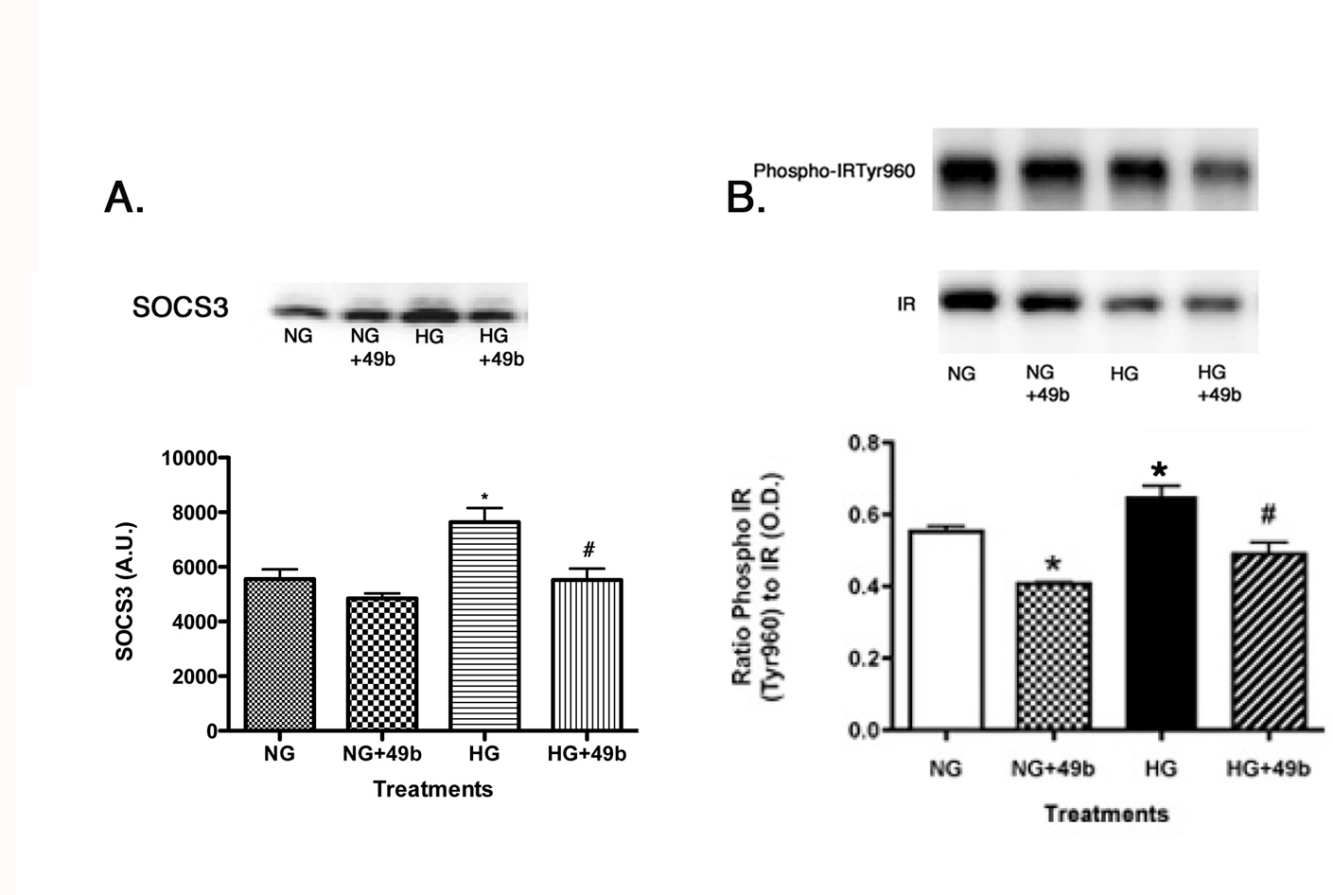
Data presented as western blot results for SOCS3 and IR^Tyr960^ on retinal Müller cells cultured in normal glucose (5 mM, NG) or high glucose (25 mM, HG) and treated with Compound 49b (50 nM). A representative blot is shown. Panel **A** is phosphorylated IR^Tyr1150/1151^ to total IR. Panel **B** is phosphorylated Akt^Ser473^ to total Akt *p<0.05 versus NG, #p<0.05 versus HG, n=4 for all groups. Data are mean±SEM.

**Figure 4:**
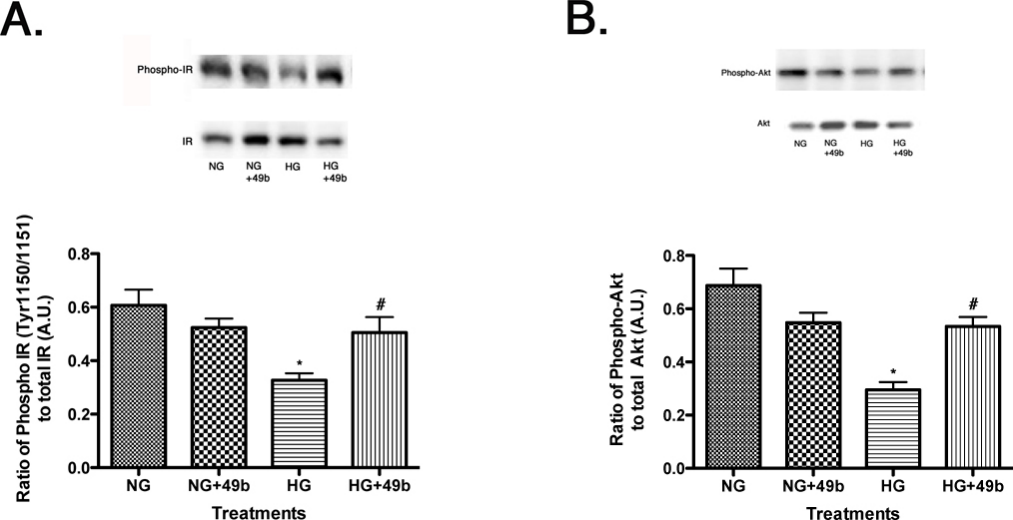
Western blotting for phosphorylated IR^Tyr1150/1151^ to total IR and phosphorylated Akt^Ser473^ to total Akt on retinal Müller cells cultured in normal glucose (5 mM, NG) or high glucose (25 mM, HG) and treated with Compound 49b (50 nM). A representative blot is shown. Panel **A** is phosphorylated IR^Tyr1150/1151^ to total IR. Panel **B** is phosphorylated Akt^Ser473^ to total Akt. *p<0.05 versus NG, #p<0.05 versus HG, n=4 for all groups. Data are mean±SEM.

**Figure 5:**
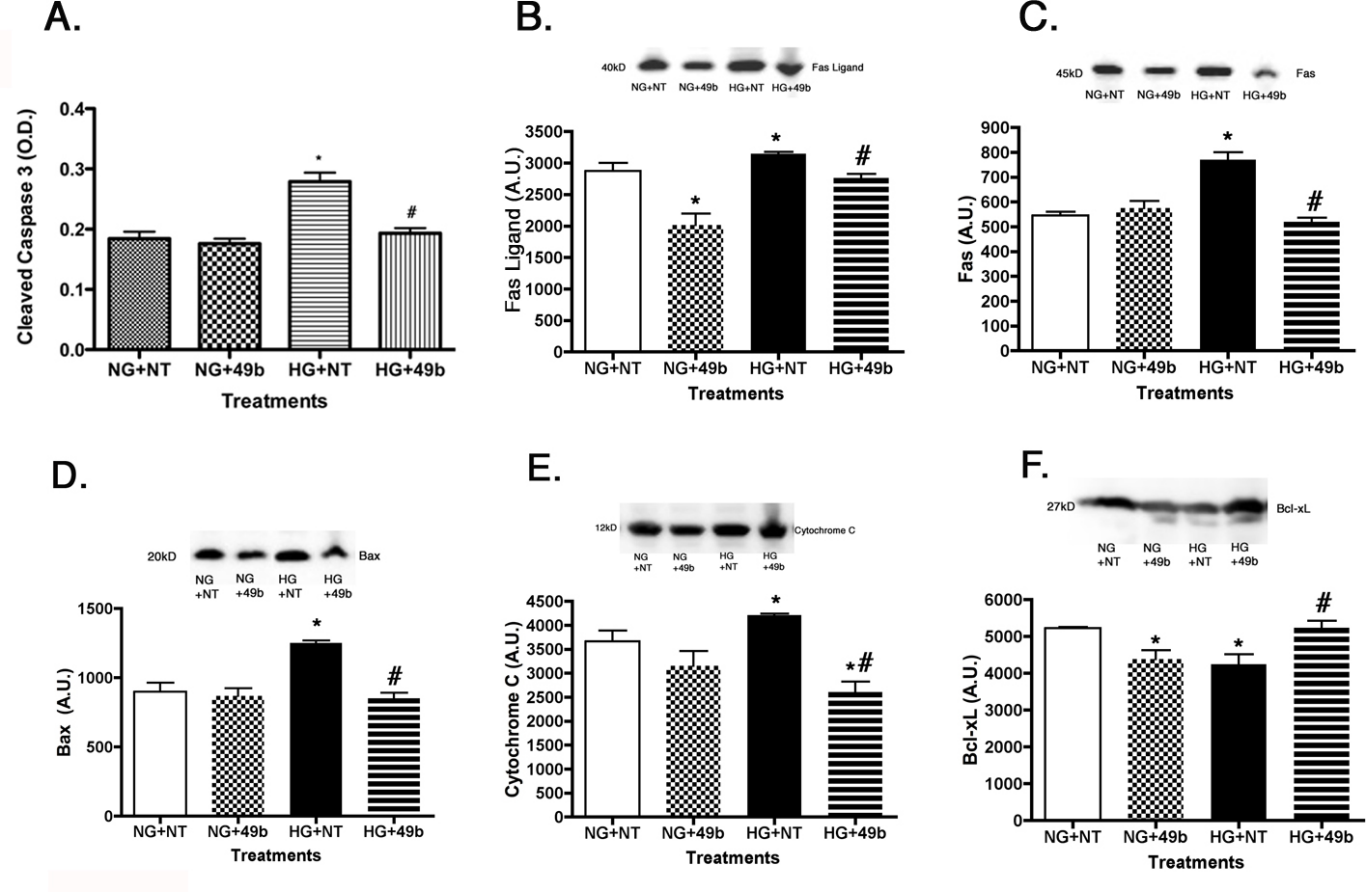
All data are for retinal Müller cells cultured in normal glucose (5 mM, NG) or high glucose (25 mM, HG) and treated with Compound 49b (50 nM). Panel **A** is ELISA results for cleaved caspase 3. Panels **B**–**F** show western blot results for Fas ligand (**B**), Fas (**C**), Bax (**D**), Cytochrome C (**E**), and Bcl-xL (**F**), to demonstrate that high glucose increases proapoptotic markers (**A**–**E**) and decreased Bcl-xL (**F**). Compound 49b increased Bcl-xL, while reducing proapoptotic factors. A representative blot is shown. *p<0.05 versus NG, #p<0.05 versus HG, n=4 for all groups. Data are mean±SEM.

**Figure 6:**
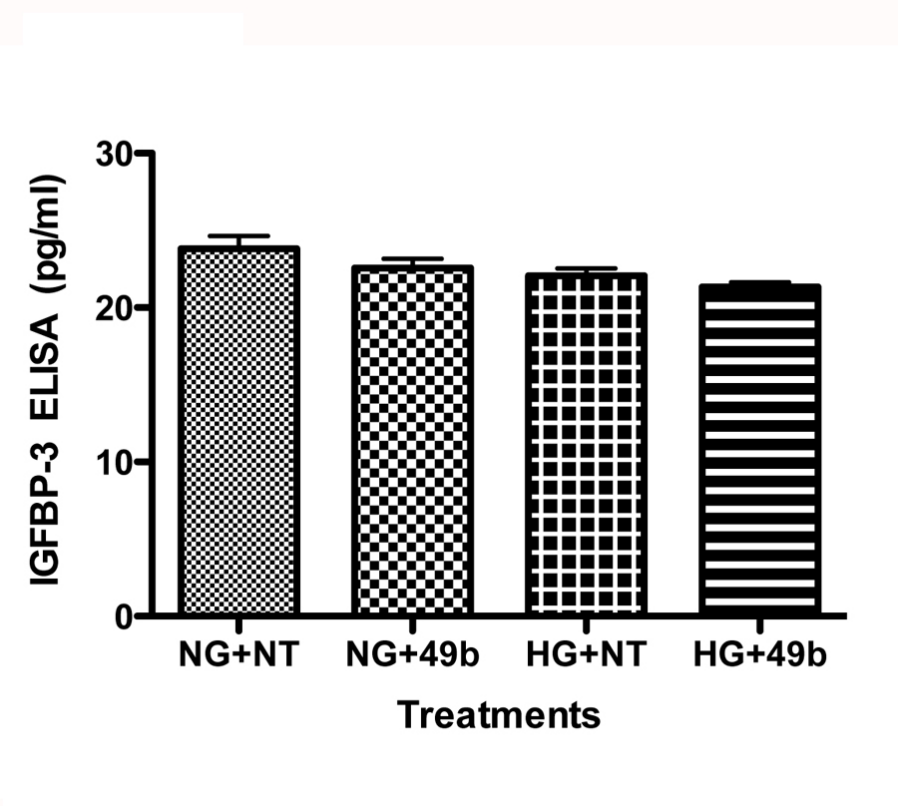
Data are ELISA results for IGFBP-3 levels for retinal Müller cells cultured in normal glucose (5 mM, NG) or high glucose (25 mM, HG) and treated with Compound 49b (50 nM). n=5 for all groups. Data are mean±SEM.

After appropriate treatments and rinsing with cold phosphate-buffered saline, retinal Müller cells were collected in lysis buffer containing the protease and phosphatase inhibitors was added to the retinal Müller cells, and lysates were scraped into tubes. Equal amounts of protein from the cell or tissue extracts were separated on the precast tris-glycine gel (Invitrogen, Carlsbad, CA), blotted onto a nitrocellulose membrane. After blocking in TBST (10 mM Tris-HCl buffer, pH 8.0, 150 mM NaCl, 0.1% Tween-20) and 5% (w/v) BSA, the membrane was treated with primary antibodies to cytochrome C, bax, Bcl-xL, IR, IR^Tyr1150/1151^, IRS-1, IRS-1^Ser307^, Akt, Akt^Ser473^, SOCS3 (Cell Signaling), Fas and Fas Ligand (Santa Cruz Biotechnology, Santa Cruz, CA) and IR^Tyr960^ (Cell Applications, San Diego, CA) at a 1:500 dilution, followed by incubation with horseradish peroxidase-labeled secondary antibodies (1:5000). The antigen-antibody complexes were detected using a chemilluminescence reagent kit (Thermo Scientific). The mean densitometry of immunoreactive bands was assessed using Kodak software Carestream Health (Rochester, NY). Results were expressed in densitometric units and compared to control groups for each individual experiment. For the phosphorylated antibodies, the ratio of phosphorylated to total protein levels are presented (Figure 1, Figure 2, Figure 3, Figure 4, Figure 5, and Figure 6).

### Statistics

All the experiments were repeated in triplicate, and the data will be presented as means ± standard error of the mean (SEM). Data were analyzed using the Kruskal-Wallis nonparametric test, followed by Dunn’s test, with p values of <0.05 considered statistically significant. In the case of western blotting, one representative blot is shown.

## Results

### Compound 49b regulated TNFα through CREB phosphorylation

As a β-adrenergic receptor agonist, Compound 49b should activate PKA, leading to CREB phosphorylation. We have previously reported that Compound 49b can regulate TNFα in RECs [1]. For this work, we wanted to demonstrate the signaling pathway by which Compound 49b regulates TNFα, which was unknown for retinal Müller cells. We demonstrated that Compound 49b increased PKA in both normal and high glucose, which is inhibited after PKA siRNA transfection (Figure 1A). As we observed in RECs, Compound 49b significantly reduced TNFα levels in retinal Müller cells, which occurred through PKA activation of CREB, as CREB siRNA plus Compound 49b eliminated Compound 49b’s inhibition of TNFα (Figure 1B). We have therefore demonstrated that β-adrenergic receptors regulated TNFα in Müller cells through the PKA-CREB pathway.

### Compound 49b reduced TNFα-mediated phosphorylation of IRS-1^Ser307^ in retinal Müller cells

Studies on adipocytes and myocytes have suggested that TNFα will increase IRS-1^Ser307^ phosphorylation in high-glucose or diabetic conditions, contributing to insulin resistance [21]. We have observed similar effects in RECs [19] and in diabetic animals [13]. In retinal Müller cells, high glucose significantly increased IRS-1^Ser307^, which is inhibited by the addition of Compound 49b (Figure 2), suggesting that Müller cell-altered insulin response may be due to TNFα actions on IRS-1.

### SOCS3 and insulin receptor^Tyr960^ regulated by Compound 49b

In addition to phosphorylating IRS-1 on serine 307, TNFα has been reported to increase SOCS3 and IR^Tyr960^, leading to blockade of normal insulin signaling in RECs [19] and adipocytes [17,22]. As expected, high glucose culturing conditions increased SOCS3 (Figure 3A) and IR^Tyr960^ levels (Figure 3B), which were significantly reduced following Compound 49b treatment to retinal Müller cells, further suggesting that β-adrenergic receptor agonists may bolster insulin signaling in the retina.

### IR^Tyr1150/1151^ and Akt phosphorylation increased after Compound 49b treatment

Normal insulin transduction occurs once insulin binds the insulin receptor→IRS-1→Akt pathway and is reduced after exposure to hyperglycemia. Compound 49b reversed this response, as we found that insulin receptor^Tyr1150/1151^ levels are increased after exposure Compound 49b (Figure 4A), leading to increased Akt phosphorylation (Figure 4B). In this manner, Compound 49b counteracted the deleterious effects of high glucose on insulin receptor signaling.

### Proapoptotic markers are decreased in retinal Müller cells cultured in high glucose following Compound 49b treatment

One of the key actions of insulin signaling is to block apoptotis of cells, thus promoting cellular homeostasis. Figure 5, shows that Compound 49b significantly decreased proapoptotic proteins (Bax, cytochrome C, Fas, Fas ligand, and cleaved caspase 3), while it increased Bcl-xL levels. This suggests that despite the high-glucose conditions, β-adrenergic receptor signaling protects retinal Müller cells.

### Compound 49b did not increase IGFBP-3 in retinal Müller cells

Many of the previous findings were to be expected, based on our previous work with salmeterol in retinal Müller cells [14,15] and work with Compound 49b in RECs [1,19]. In RECs, the key antiapoptotic pathway activated by Compound 49b involves activation of insulin-like IGFBP-3. Figure 6 demonstrates that while much of the signaling is similar, retinal Müller cell response to Compound 49b does not involve IGFBP-3. This follows from our previous findings that RECs were not highly involved in insulin receptor signaling; rather, RECs appeared to respond to IGF-1 activation for signaling. This suggests that β-adrenergic adrenergic receptors are protective against apoptosis in both RECs and retinal Müller cells; however, the signaling pathways mediating the protection are different.

## Discussion

Rates of type 2 diabetes are expected to reach epidemic levels before 2030. This will be associated with a significant increase in the number of patients with diabetes complications, notably diabetic retinopathy. While work on insulin resistance in the retina is sparse, much more has been done on other targets, such as adipocytes and myocytes [23]. TNFα suppresses the insulin response in HepG2 cells [24]. Endothelial cells within the brown adipose tissue of rats have significantly increased levels of TNFα, which was associated with increased endothelial cell apoptosis [25]. In addition to the studies on rodents, work in humans with artherosclerosis demonstrated that significantly increased serum TNFα levels in patients were associated with impaired glucose tolerance and type 2 diabetes, after correcting for other variables [26]. In the retina, levels of systemic soluble TNFα receptors 1 and 2 were increased in Hispanic patients with diabetic retinopathy [27]. Levels of TNFα in these Hispanic patients were not measured. Taken together, there is an abundance of support for the conclusion that TNFα levels are associated with insulin resistance, although the mechanism in the retina is less clear.

The next step to determining whether TNFα is key to insulin resistance is to evaluate whether TNFα can regulate other factors involved in impaired insulin signaling. We have previously demonstrated that TNFα can increase SOCS3 levels in RECs [19], and this also occurs in retinal Müller cells. Work in rats with intrauterine growth retardation showed increased SOCS3 levels, which impaired insulin signaling in hepatocytes [28]. In the rats with growth retardation, use of SOCS3 siRNA on cultured hepatocytes from the rats significantly improved insulin receptor signal transduction [28]. Using a Cre-Lox expression system in mice to detect adipose tissue, researchers showed that knockout of SOCS3 lowered SOCS3 levels, specifically in the adipose tissue, and that it was associated with improved insulin sensitivity and enhanced phosphorylation of IRS-1 [29], thus improving insulin signaling. Similar to the work on adipose tissue, studies also showed that elimination of SOCS3 from the skeletal muscle increased phosphorylation of IRS-1 and Akt [30], again suggesting that TNFα activation of SOCS3 is key to impaired insulin sensitivity.

Data from other targets suggest that hyperglycemia-induced TNFα can activate SOCS3, leading to impaired insulin signaling. We find similar results in retinal Müller cells, where high glucose culture conditions lead to increased TNFα and SOCS3 levels, with increased phosphorylation of IRS-1^Ser307^ and IR^Tyr960^. These negative changes to normal insulin signal transduction are associated with increased apoptosis of retinal Müller cells. In contrast to our previous work on RECs, where IGFBP-3 was key to reduced apoptosis, IGFBP-3 levels are not altered in retinal Müller cells. Compound 49b can reduce all markers of insulin resistance in the retinal Müller cells, suggesting it is protective of the retina.

While we have previously reported that a specific β-2-adrenergic receptor agonist can regulate TNFα and insulin signaling in retinal Müller cells [14,15], this work did not investigate SOCS3 or IR^Tyr960^ signaling. Similarly, RECs in high glucose have altered TNFα and SOCS3 levels; however, no treatments were assessed in that study [19]. Because we previously reported that IGFBP-3 is key to apoptosis in RECs [1] and that β-adrenergic receptors and insulin signaling regulate REC apoptosis in independent pathways [31], we felt it important to demonstrate that a novel β-adrenergic receptor agonist, with both β-1 and β-2-adrenergic receptor actions, could improve insulin sensitivity in retinal Müller cells through decreased TNFα levels. These studies lay the groundwork for additional studies on the specific phosphorylation events of IRS-1 and the insulin receptor by TNFα and SOCS3 during high glucose exposure in retinal Müller cells. Additionally, since Müller cells are one of the first responders to stress in the retina, additional understanding of this response to stress and insulin response is potentially key to the diabetic changes in the retina.

Taken together, these studies demonstrate that high glucose leads to impaired insulin signal transduction in retinal Müller cells. Increased TNFα leads to increased SOCS3 levels, which impairs normal insulin→IRS-1→Akt signaling. Compound 49b can reduce TNFα levels, leading to increased insulin receptor and Akt phosphorylation, resulting in reduced apoptosis.
